# 5,7-Dichloro­quinolin-8-ol

**DOI:** 10.1107/S1600536809014846

**Published:** 2009-04-25

**Authors:** Seik Weng Ng

**Affiliations:** a Department of Chemistry, University of Malaya, 50603 Kuala Lumpur, Malaysia

## Abstract

The mol­ecule of the title compound, C_9_H_5_Cl_2_NO, is essentially planar [give maximum or r.m.s. deviation] and the hydr­oxy group acts as a hydrogen-bond donor to the N atom of a symmetry-related mol­ecule, generating a hydrogen-bonded dimer,which lies on a twofold rotation axis.

## Related literature

Unlike quinolin-8-ol, which yields a large number of metal derivatives, 5,7-dichloro­quinolin-8-ol forms only a small number of metal chelates. For their crystal structures, see: García-Granda *et al.* (1987 ▶); Artizzu *et al.* (2007 ▶, 2008 ▶); Day *et al.* (1980 ▶); González-Baró *et al.* (1998 ▶); Horton & Wendlandt (1963 ▶); Miyashita *et al.* (2005 ▶); Suganuma *et al.* (2001 ▶); Van Deun *et al.* (2004 ▶).
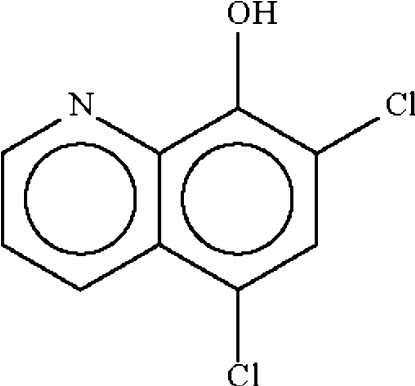


## Experimental

### 

#### Crystal data

C_9_H_5_Cl_2_NO
*M* *_r_* = 214.04
Monoclinic, 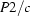

*a* = 15.5726 (3) Å
*b* = 3.8062 (1) Å
*c* = 16.1269 (3) Å
β = 118.029 (1)°
*V* = 843.76 (3) Å^3^
*Z* = 4
Mo *K*α radiation
μ = 0.72 mm^−1^
*T* = 123 K
0.36 × 0.09 × 0.02 mm

#### Data collection

Bruker SMART APEX diffractometer
Absorption correction: multi-scan (*SADABS*; Sheldrick, 1996 ▶) *T*_min_ = 0.782, *T*_max_ = 0.986
7279 measured reflections
1919 independent reflections
1644 reflections with *I* > 2σ(*I*)
*R*_int_ = 0.032

#### Refinement

*R*[*F* ^2^ > 2σ(*F* ^2^)] = 0.040
*wR*(*F* ^2^) = 0.112
*S* = 1.05
1919 reflections
122 parameters
1 restraint
H atoms treated by a mixture of independent and constrained refinement
Δρ_max_ = 0.55 e Å^−3^
Δρ_min_ = −0.36 e Å^−3^

### 

Data collection: *APEX2* (Bruker, 2008 ▶); cell refinement: *SAINT* (Bruker, 2008 ▶); data reduction: *SAINT*; program(s) used to solve structure: *SHELXS97* (Sheldrick, 2008 ▶); program(s) used to refine structure: *SHELXL97* (Sheldrick, 2008 ▶); molecular graphics: *X-SEED* (Barbour, 2001 ▶); software used to prepare material for publication: *publCIF* (Westrip, 2009 ▶).

## Supplementary Material

Crystal structure: contains datablocks global, I. DOI: 10.1107/S1600536809014846/lh2808sup1.cif

Structure factors: contains datablocks I. DOI: 10.1107/S1600536809014846/lh2808Isup2.hkl

Additional supplementary materials:  crystallographic information; 3D view; checkCIF report

## Figures and Tables

**Table 1:** Hydrogen-bond geometry (Å, °)

*D*—H⋯*A*	*D*—H	H⋯*A*	*D*⋯*A*	*D*—H⋯*A*
O1—H1⋯N1^i^	0.84 (1)	2.01 (2)	2.761 (2)	150 (3)

Symmetry code: (i) 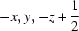.

**Fig. 1.**
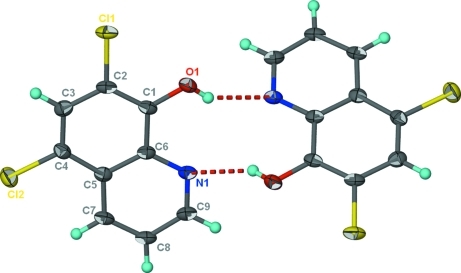
Thermal ellipsoid plot (Barbour, 2001) of a hydrogen-bonded dimer of the title compound; ellipsoids are drawn at the 70% probability level and H atoms of arbitrary radius. The unlabeled molecule is related by the symmetry operator -x, y, -z+1/2

## supplementary crystallographic information

### Comment

A hydrogen-bonded dimer of the title compound is shown in Fig. 1.

### Experimental

The organic reactant was returned unchanged in an unsuccessful attempt at reacting it with a zinc salt in methanol.

### Refinement

Carbon-bound H-atoms were placed in calculated positions (C–H 0.93 Å) and were included in the refinement in the riding model approximation, with *U*(H) set to 1.2 *U*(C). The hydroxy hydrogen atom was located in a difference Fourier map, and was refined with a distance restraint of O–H 0.84±0.01 Å; its temperature factor was freely refined.

### Crystal data

**Table:**

C_9_H_5_Cl_2_NO	*F*(000) = 432
*M**_r_* = 214.04	*D*_x_ = 1.685 Mg m^−^^3^
Monoclinic, *P*2/*c*	Mo *K*α radiation, λ = 0.71073 Å
Hall symbol: -P 2yc	Cell parameters from 3573 reflections
*a* = 15.5726 (3) Å	θ = 2.5–28.3°
*b* = 3.8062 (1) Å	µ = 0.72 mm^−^^1^
*c* = 16.1269 (3) Å	*T* = 123 K
β = 118.029 (1)°	Plate, colorless
*V* = 843.76 (3) Å^3^	0.36 × 0.09 × 0.02 mm
*Z* = 4	

### Data collection

**Table:**

Bruker SMART APEX diffractometer	1919 independent reflections
Radiation source: fine-focus sealed tube	1644 reflections with *I* > 2σ(*I*)
graphite	*R*_int_ = 0.032
ω scans	θ_max_ = 27.5°, θ_min_ = 1.5°
Absorption correction: multi-scan (*SADABS*; Sheldrick, 1996)	*h* = −20→20
*T*_min_ = 0.782, *T*_max_ = 0.986	*k* = −4→4
7279 measured reflections	*l* = −20→20

### Refinement

**Table:**

Refinement on *F*^2^	Primary atom site location: structure-invariant direct methods
Least-squares matrix: full	Secondary atom site location: difference Fourier map
*R*[*F*^2^ > 2σ(*F*^2^)] = 0.040	Hydrogen site location: inferred from neighbouring sites
*wR*(*F*^2^) = 0.112	H atoms treated by a mixture of independent and constrained refinement
*S* = 1.05	*w* = 1/[σ^2^(*F*_o_^2^) + (0.0684*P*)^2^ + 0.4949*P*] where *P* = (*F*_o_^2^ + 2*F*_c_^2^)/3
1919 reflections	(Δ/σ)_max_ < 0.001
122 parameters	Δρ_max_ = 0.55 e Å^−^^3^
1 restraint	Δρ_min_ = −0.36 e Å^−^^3^

### Special details

**Table:**

Geometry. All e.s.d.'s (except the e.s.d. in the dihedral angle between two l.s. planes) are estimated using the full covariance matrix. The cell e.s.d.'s are taken into account individually in the estimation of e.s.d.'s in distances, angles and torsion angles; correlations between e.s.d.'s in cell parameters are only used when they are defined by crystal symmetry. An approximate (isotropic) treatment of cell e.s.d.'s is used for estimating e.s.d.'s involving l.s. planes.

### Fractional atomic coordinates and isotropic or equivalent isotropic displacement parameters (Å^2^)

**Table:**

	*x*	*y*	*z*	*U*_iso_*/*U*_eq_	
Cl1	0.29367 (4)	0.38966 (13)	0.24587 (3)	0.02310 (17)	
Cl2	0.44255 (4)	0.89258 (14)	0.59720 (3)	0.02635 (18)	
O1	0.10638 (11)	0.6233 (4)	0.22642 (10)	0.0223 (3)	
N1	0.07459 (12)	0.9357 (4)	0.36439 (11)	0.0186 (4)	
H1	0.0565 (14)	0.729 (7)	0.220 (2)	0.051 (9)*	
C1	0.18119 (14)	0.6852 (5)	0.31223 (13)	0.0176 (4)	
C2	0.27416 (15)	0.5885 (5)	0.33230 (13)	0.0184 (4)	
C3	0.35486 (14)	0.6504 (5)	0.42003 (14)	0.0193 (4)	
H3	0.4180	0.5794	0.4316	0.023*	
C4	0.34192 (14)	0.8134 (5)	0.48869 (13)	0.0183 (4)	
C5	0.24865 (14)	0.9212 (5)	0.47354 (13)	0.0168 (4)	
C6	0.16747 (14)	0.8502 (5)	0.38470 (13)	0.0170 (4)	
C7	0.23008 (15)	1.0924 (5)	0.54108 (13)	0.0191 (4)	
H7	0.2822	1.1462	0.6013	0.023*	
C8	0.13659 (15)	1.1807 (5)	0.51946 (14)	0.0201 (4)	
H8	0.1230	1.2970	0.5641	0.024*	
C9	0.06103 (15)	1.0957 (5)	0.42978 (14)	0.0204 (4)	
H9	−0.0036	1.1574	0.4155	0.024*	

### Atomic displacement parameters (Å^2^)

**Table:**

	*U*^11^	*U*^22^	*U*^33^	*U*^12^	*U*^13^	*U*^23^
Cl1	0.0336 (3)	0.0238 (3)	0.0188 (3)	0.00576 (19)	0.0180 (2)	0.00172 (18)
Cl2	0.0229 (3)	0.0305 (3)	0.0203 (3)	0.0002 (2)	0.0057 (2)	−0.00406 (19)
O1	0.0229 (7)	0.0304 (8)	0.0137 (6)	0.0030 (6)	0.0086 (6)	−0.0021 (5)
N1	0.0230 (8)	0.0202 (9)	0.0157 (7)	0.0008 (6)	0.0117 (6)	0.0020 (6)
C1	0.0242 (10)	0.0166 (9)	0.0139 (8)	−0.0012 (7)	0.0105 (7)	0.0016 (7)
C2	0.0280 (10)	0.0158 (10)	0.0171 (9)	0.0009 (7)	0.0154 (8)	0.0016 (7)
C3	0.0223 (9)	0.0173 (10)	0.0215 (9)	0.0015 (7)	0.0130 (8)	0.0025 (8)
C4	0.0211 (9)	0.0182 (10)	0.0145 (8)	−0.0019 (7)	0.0074 (7)	0.0014 (7)
C5	0.0216 (9)	0.0142 (9)	0.0164 (9)	−0.0012 (7)	0.0104 (7)	0.0021 (7)
C6	0.0220 (9)	0.0161 (9)	0.0162 (9)	−0.0010 (7)	0.0117 (7)	0.0010 (7)
C7	0.0276 (10)	0.0183 (10)	0.0143 (8)	−0.0029 (8)	0.0122 (8)	−0.0001 (7)
C8	0.0297 (10)	0.0187 (10)	0.0181 (9)	−0.0013 (8)	0.0164 (8)	−0.0007 (7)
C9	0.0263 (10)	0.0210 (10)	0.0200 (9)	0.0004 (8)	0.0160 (8)	0.0019 (7)

### Geometric parameters (Å, °)

**Table:**

Cl1—C2	1.7337 (19)	C3—H3	0.9500
Cl2—C4	1.7412 (19)	C4—C5	1.416 (3)
O1—C1	1.346 (2)	C5—C7	1.411 (3)
O1—H1	0.835 (10)	C5—C6	1.422 (3)
N1—C9	1.318 (2)	C7—C8	1.370 (3)
N1—C6	1.364 (2)	C7—H7	0.9500
C1—C2	1.377 (3)	C8—C9	1.408 (3)
C1—C6	1.428 (3)	C8—H8	0.9500
C2—C3	1.402 (3)	C9—H9	0.9500
C3—C4	1.364 (3)		
			
C1—O1—H1	111 (2)	C7—C5—C6	117.26 (17)
C9—N1—C6	117.95 (17)	C4—C5—C6	118.24 (17)
O1—C1—C2	120.07 (17)	N1—C6—C5	122.49 (17)
O1—C1—C6	121.87 (17)	N1—C6—C1	117.25 (17)
C2—C1—C6	118.05 (17)	C5—C6—C1	120.26 (17)
C1—C2—C3	122.49 (18)	C8—C7—C5	119.71 (18)
C1—C2—Cl1	119.33 (15)	C8—C7—H7	120.1
C3—C2—Cl1	118.17 (15)	C5—C7—H7	120.1
C4—C3—C2	119.43 (18)	C7—C8—C9	118.71 (17)
C4—C3—H3	120.3	C7—C8—H8	120.6
C2—C3—H3	120.3	C9—C8—H8	120.6
C3—C4—C5	121.50 (18)	N1—C9—C8	123.88 (18)
C3—C4—Cl2	119.21 (15)	N1—C9—H9	118.1
C5—C4—Cl2	119.28 (14)	C8—C9—H9	118.1
C7—C5—C4	124.50 (18)		
			
O1—C1—C2—C3	179.21 (17)	C7—C5—C6—N1	−1.1 (3)
C6—C1—C2—C3	−0.9 (3)	C4—C5—C6—N1	178.25 (17)
O1—C1—C2—Cl1	0.4 (3)	C7—C5—C6—C1	178.50 (17)
C6—C1—C2—Cl1	−179.66 (14)	C4—C5—C6—C1	−2.1 (3)
C1—C2—C3—C4	−0.2 (3)	O1—C1—C6—N1	1.6 (3)
Cl1—C2—C3—C4	178.56 (15)	C2—C1—C6—N1	−178.29 (17)
C2—C3—C4—C5	0.2 (3)	O1—C1—C6—C5	−178.02 (17)
C2—C3—C4—Cl2	−179.52 (14)	C2—C1—C6—C5	2.1 (3)
C3—C4—C5—C7	−179.67 (18)	C4—C5—C7—C8	−179.00 (19)
Cl2—C4—C5—C7	0.0 (3)	C6—C5—C7—C8	0.3 (3)
C3—C4—C5—C6	1.0 (3)	C5—C7—C8—C9	0.3 (3)
Cl2—C4—C5—C6	−179.31 (14)	C6—N1—C9—C8	−0.6 (3)
C9—N1—C6—C5	1.2 (3)	C7—C8—C9—N1	−0.2 (3)
C9—N1—C6—C1	−178.39 (17)		

### Hydrogen-bond geometry (Å, °)

**Table:**

*D*—H···*A*	*D*—H	H···*A*	*D*···*A*	*D*—H···*A*
O1—H1···N1^i^	0.84 (1)	2.01 (2)	2.761 (2)	150 (3)

Symmetry codes: (i) −*x*, *y*, −*z*+1/2.
